# Differential response of luminal and basal breast cancer cells to acute and chronic hypoxia

**DOI:** 10.1007/s10549-023-06863-w

**Published:** 2023-02-24

**Authors:** Qiuyu Liu, Nasi Liu, Vera van der Noord, Wanda van der Stel, Bob van de Water, Erik H. J. Danen, Sylvia E. Le Dévédec

**Affiliations:** Division of Drug Discovery and Safety, Leiden Academic Centre of Drug Research, Leiden University, Leiden, The Netherlands

**Keywords:** Breast cancer, Acute hypoxia, Chronic hypoxia, Luminal, Basal

## Abstract

Hypoxia is linked to disease progression and poor prognosis in several cancers, including breast cancer. Cancer cells can encounter acute, chronic, and/or intermittent periods of oxygen deprivation and it is poorly understood how the different breast cancer subtypes respond to such hypoxia regimes. Here, we assessed the response of representative cell lines for the luminal and basal A subtype to acute (24 h) and chronic hypoxia (5 days). High throughput targeted transcriptomics analysis showed that HIF-related pathways are significantly activated in both subtypes. Indeed, HIF1⍺ nuclear accumulation and activation of the HIF1⍺ target gene CA9 were comparable. Based on the number of differentially expressed genes: (i) 5 days of exposure to hypoxia induced a more profound transcriptional reprogramming than 24 h, and (ii) basal A cells were less affected by acute and chronic hypoxia as compared to luminal cells. Hypoxia-regulated gene networks were identified of which hub genes were associated with worse survival in breast cancer patients. Notably, while chronic hypoxia altered the regulation of the cell cycle in both cell lines, it induced two distinct adaptation programs in these subtypes. Mainly genes controlling central carbon metabolism were affected in the luminal cells whereas genes controlling the cytoskeleton were affected in the basal A cells. In agreement, in response to chronic hypoxia, lactate secretion was more prominently increased in the luminal cell lines which were associated with the upregulation of the GAPDH glycolytic enzyme. This was not observed in the basal A cell lines. In contrast, basal A cells displayed enhanced cell migration associated with more F-actin stress fibers whereas luminal cells did not. Altogether, these data show distinct responses to acute and chronic hypoxia that differ considerably between luminal and basal A cells. This differential adaptation is expected to play a role in the progression of these different breast cancer subtypes.

**Supplementary Information:**

The online version contains supplementary material available at 10.1007/s10549-023-06863-w.

## Introduction

Breast cancer remains the second leading cause of mortality in women [1]. Breast cancer can be divided into different subtypes including luminal A and B, basal-like (basal A and B), and HER-2 positive. Altered metabolism and increased invasiveness are two hallmarks of cancer, which both contribute to cancer progression and cancer cell crosstalk [2, 3]. Hypoxia, a key feature in growing solid tumors regulates both hallmarks and is therefore associated with an aggressive phenotype of breast cancer.

Hypoxia controls the expression of hundreds of genes involved in critical processes in breast malignancy such as metabolism [4, 5], cell motility [6], and epithelial-to-mesenchymal transition (EMT) [7]. This transcriptional regulation is tightly orchestrated by hypoxia-inducible factors (HIFs), transcription factors whose activity is sensitive to hypoxic stress [8]. HIFs comprising an alpha subunit oxygen-sensitive) and beta subunit (constitutively expressed) bind to hypoxia response elements (HREs) in the genome, regulating the cellular response to oxygen deprivation [9]. The tissue microenvironment of breast cancer is highly heterogenous and, depending on the local extent of newly formed blood vessels, it can contain regions of acute, intermittent and chronic hypoxia [10]. This is relevant as cancer cells show different cellular responses depending on the duration and frequency of hypoxia exposure [9, 11, 12]. Acute hypoxia is associated with reversible changes, chronic hypoxia is associated with long-term cellular changes potentially developing into mutagenesis and genetic instability [9].

The function of HIFs depends on both the level of oxygen and the duration of the hypoxic insult, and, consequently, their activities vary between acute and chronic hypoxia [8, 10, 13]. In addition, the type of hypoxia also influences the response of the microenvironment. For example, acute hypoxia leads to endothelial cell death and microvascular permeability associated with increased lung metastasis of breast cancer cells whereas chronic hypoxia ultimately stimulates endothelial cell proliferation and vascular integrity which delays metastasis [14]. These findings indicate that the duration of hypoxic insults may represent an important factor in the progression of breast cancer that has not been fully explored. In addition, it is poorly understood if different breast cancer subtypes may be differently influenced by hypoxia.

To address these questions, in this study we focused on the luminal and basal A subtypes that share a predominantly epithelial morphology, in contrast to triple-negative breast cancer (TNBC) cells that are typically more mesenchymal [15, 16]. Hypoxia-induced EMT is associated with increased cell motility [17] and high glycolytic activity [18], and TNBC cells already display such EMT features. Using transcriptomics analysis, we identify shared and distinct responses to acute versus chronic hypoxia for luminal versus basal cells. Most notably, an initial shared HIF1α- response to acute hypoxia is followed by distinct metabolic versus migratory responses to chronic hypoxia in luminal and basal cells, respectively. Our findings shed new light on the responses of different breast cancer subtypes to different regimes of hypoxia that may point to actionable mechanisms driving disease progression.

## Material and methods

### Cell culture

Human breast cancer cell lines (luminal: MCF-7, T47D, BT474; basal A: SUM149PT, HCC1806, and HCC1143) were obtained from American Type Culture Collection (ATCC). Cells were cultured in RPMI-1640 medium (#52400; Gibco, Waltham, MA, USA) with 10% FBS, 25U/ml penicillin, and 25 µg/ml streptomycin (#15140122; Gibco) in an incubator at 37 °C with 5% CO_2_ and 21% O_2_ (normoxia) or 1% O_2_ (hypoxia).

### Human whole transcriptome analysis (TempO-Seq)

Targeted whole transcriptome analysis was performed using TempO-Seq [19] (BioSpyder Technologies Inc., Carlsbad, CA, USA). 3000–7000 cells/well of MCF-7 and HCC1143 were seeded in 96-well plates. Cells were lysed in 1 × BioSpyder lysis buffer after 24 h (https://www.ebi.ac.uk/biostudies/arrayexpress/studies/E-MTAB-12234?key=06b3e420-93c3-4fec-86dc-11c2d688e71c) or 5 days incubation (https://www.ebi.ac.uk/biostudies/arrayexpress/studies/E-MTAB-12233?key=5b1f9227-fe06-44b4-8909-55bb3ecf7a33) under normoxia or hypoxia. Samples from three biological replicates were stored at −80 °C and shipped to BioSpyder for TempO-Seq analysis. An in-house developed R script was used for count normalization and differential gene expression analysis. The minimum library size (total number of reads per sample) was set as 100.000 reads and samples below this size were removed. The DESeq2 package was used for count data normalization and to generate adjusted *p*-value (padj) and log_2_FoldChange values. Differentially expressed genes (DEGs) were selected by |log_2_Foldchange|> 1 and padj < 0.05. Given the high number of DEGs in the HCC1143 5 days dataset, in this case, |log_2_Foldchange|> 2 and padj < 0.01 was used for subsequent further analysis using bioinformatics tools. Heatmaps and Venn diagrams were made using OmicStudio tools at https://www.omicstudio.cn/tool.

### Immunofluorescence

Cells were fixed with 4% paraformaldehyde and 0.3% Triton X-100, washed with PBS, blocked with 0.5% Bovine Serum Albumin (BSA) for 30 min at room temperature, and stained with HIF1⍺ antibody (#610,959; BD Biosciences, Franklin Lakes, NJ, USA) and Rhodamine Phalloidin (R415; Thermo Fisher Scientific, Waltham, MA, USA) overnight at 4 °C and 2 h at RT, respectively. Cells were rinsed with 0.5% BSA every 10 min 3 times and stained with secondary antibody and Hoechst 33,258 (#610959; Thermo Fisher) for 1 h at room temperature (RT) in the dark. After washing with PBS, plates were stored at 4 °C. Images were taken with a Nikon Eclipse Ti microscope with a 20 × objective. Scale bars shown in the images represent 50 µm.

### Western blotting

MCF-7 and HCC1143 cells were seeded at densities leading to sub confluency at the time of analysis and cultured under normoxia and hypoxia for 24 h and 5 days. Then, cells were lysed with RIPA buffer containing 1% proteases/phosphatases inhibitor cocktail (P8340; Sigma-Aldrich, Burlington, MA, USA). The protein concentration was measured by BCA assay. Protein lysates were separated by SDS-PAGE and transferred to PVDF membranes. Membranes were blocked with 5% BSA and incubated with Carbonic Anhydrase IX (CA9) antibody (5649S; Cell Signaling Technology,

Danvers, MA, USA), PLOD2 antibody (MAB4445; R&D Systems, Minneapolis, MN, USA), glyceraldehyde 3-phosphate dehydrogenase (GAPDH) antibody (sc-32233; Santa Cruz, Dallas, TX, USA), or β-actin antibody (sc-47778; Santa Cruz) overnight at 4 °C. Membranes were washed and incubated with HRP- or Cy5-conjugated secondary antibodies and signals were detected with an Amersham Imager (GE Healthcare Life Science, Chicago, IL, USA). Quantification of bands was done by Image J.

### RT-qPCR

Total RNA was isolated by RNeasy Plus Mini Kit (#74,136; Qiagen, Hilden, Germany) and cDNA was synthesized by the RevertAid H Minus First Strand cDNA Synthesis Kit (Thermo Fisher Scientific, Waltham, MA, USA). qPCR was performed using SYBR Green PCR master mix (Thermo Fisher Scientific) on a Real-Time PCR instrument (Applied Biosystems, Waltham, MA, USA). The following qPCR primer sets were used: β-actin forward (fw), 5′- ATTGCCGACAGGATGCAGAA -3′; β-actin reverse (rev), 5′- GCTGATCCACATCTGCTGGAA-3′; CA9 forward (fw), 5′- CATCCTAGCCCTGGTTTTTGG-3′; CA9 reverse (rev), 5′- GCTCACACCCCTTTGGTT-3′; PLOD2 forward (fw), 5′- GCGTTCTCTTCGTCCTCATCA-3′; PLOD2 reverse (rev), 5′- TGAAGCTCCAGCCTTTTCGTG-3′. Relative gene expression was calculated with the 2^–∆∆CT^ method.

### Lactate assay

Cells were cultured under normoxia and hypoxia for 24 h and 5 days, and supernatant media were collected. 10 µl of the supernatant medium was added into 96-well plates in each well, and 90 μl of reagent mix (80% TRAM buffer (108 mM Triethanolamine HCl (T9534; Sigma, Burlington, MA, USA), 10.7 mM EDTA-Na2 (E4884; Merck, Burlington, MA, USA), 42 mM MgCl2 (M8266; Merck) in ddH20, pH 7.5.), 20% color reagent (1.63 mM PMS (P9625; Merck), 3.95 mM INT (I8377; Merck), 35% ethanol, and 2% Triton X-100 (T8787; Sigma-Aldrich)), 3.3 mM β-NAD (N7004; Merck) and 0.33 μl/ml LDH (L2500; Merck)) was added. Plates were incubated for 7 min at RT avoiding light. Optical density was measured in a plate reader at 490 nm and lactate was quantified against a lactate standard curve (#71718; Fluka Chemika, Buchs, Switzerland) using the abc-formula (ax^2^ + bx + c = 0).

### Sulforhodamine B (SRB) assay

Plates used for lactate assay were subsequently fixed with 50% TCA for 1 h at 4 °C, washed with demi-water, and air dried at RT. 0.4% SRB was added to plates and incubated for 2 h avoiding light at RT. Plates were washed with 1% acetic acid at least 5 times every hour to get rid of unbound SRB and air dried. 10 mM Tris was added in the wells to dissolve the bound SRB and the plates were gently shaken for 2 h at RT gentle. The absorbance was measured with a plate reader (Tecan Infinite M1000, Männedorf, Switzerland).

### Cell migration quantification assay

3000–7000 cells/well of MCF-7 and HCC1143 were seeded onto 20 µg/ml collagen-coated 96-well plates. After 24 h or 5 days under normoxia or hypoxia medium was supplemented with 1:10,000 Hoechst (#33242; Thermo Fisher) and images were taken every 10 min using a Nikon Eclipse Ti microscope and a 20 × air objective. The hypoxic incubator on the microscope allowed an O_2_ controlled environment at 21% O_2_ or 1% O_2_. Image analysis was performed using in-house developed Cell Profiler [20] and R [21] pipelines.

### siRNA-mediated gene knockdown

50 nM SMARTpool siRNA (Dharmacon, Lafayette, CO, USA) was transfected into cells using INTERFERin (Polyplus, Illkirch-Strasbourg, France). A KinasePool mixture containing > 100 siRNAs targeting kinases with a total siRNA concentration of 50 nM (i.e., 0.5 nM of each siRNA) was used as control. 3000–7000 cells/well of MCF-7 and HCC1143 were seeded in 96-well plates and 8 × 10^4^–10 × 10^4^ cells/ well were seeded in 12-well plates. The medium was refreshed after 18 h.

### Cell cycle analysis using FUCCI reporter cell lines

MCF-7 and HCC1143 cells were transfected with pLL3.7 m-Clover-Geminin (1–110)-IRES-mKO2-Cdt (30–120) (#83,841; Addgene, Watertown, MA, USA). Transfected cells were enriched by fluorescence-activated cell sorting (FACS) after 1-week of cell culture using a cell sorter (Sony, SH800S Cell Sorter, San Jose, CA, USA). Medium was supplemented with 1:10,000 Hoechst (#33,242; Thermo Fisher) and images were taken every 10 min using a Nikon Eclipse Ti microscope and O_2_ controlled environment at 21% O_2_ or at 1% O_2_ using a 20 × objective. Quantification of cell cycle phases in MCF-7 and HCC1143 FUCCI reporter cell lines was performed with CellProfiler.

### Bioinformatics analysis of gene functional enrichment and annotation

Enrichment analysis was performed on the Metascape platform (https://metascape.org/gp/index.html#/main/step1) [22]. Pathway analysis was performed by Ingenuity Pathway Analysis software (IPA; QIAGEN), and in R with the clusterProfiler package using the Kyoto Encyclopedia of Genes and Genomes (KEGG) database. Protein–protein interaction (PPI) networks were analyzed using STRING database (https://string-db.org/). The *cytoHubba* plugin in Cytoscape software (version 3.7.2) was used to identify hub genes and their networks. The “Degree” algorithm was used to select the top genes in Cytoscape.

### Analysis of clinical associations of hub genes

The association of the mean expression of the hub genes in each category selected from Cytoscape (gene networks derived from DEGs in acute or chronic hypoxia in MCF-7 or HCC1143 cells) in breast cancer patients with relapse-free survival (RFS) was analyzed using the Kaplan–Meier (KM) plotter platform (https://kmplot.com/analysis/). Logrank *p*-value < 0.01 was considered significant for the difference between high and low expressors.

### Statistical analysis

All statistical analysis was performed using GraphPad Prism 9 using t-test or one-way ANOVA. All experiments were performed with at least three biological replicates where not indicated. Data were expressed as mean $\pm$ SD. Significance was indicated by **** (*p* < 0.0001), *** (*p* < 0.001), ** (*p* < 0.01), * (*p* < 0.05), and ns (not significant).

## Results

### Luminal and basal A breast cancer cells respond to acute and chronic hypoxia

**Fig. 1:**
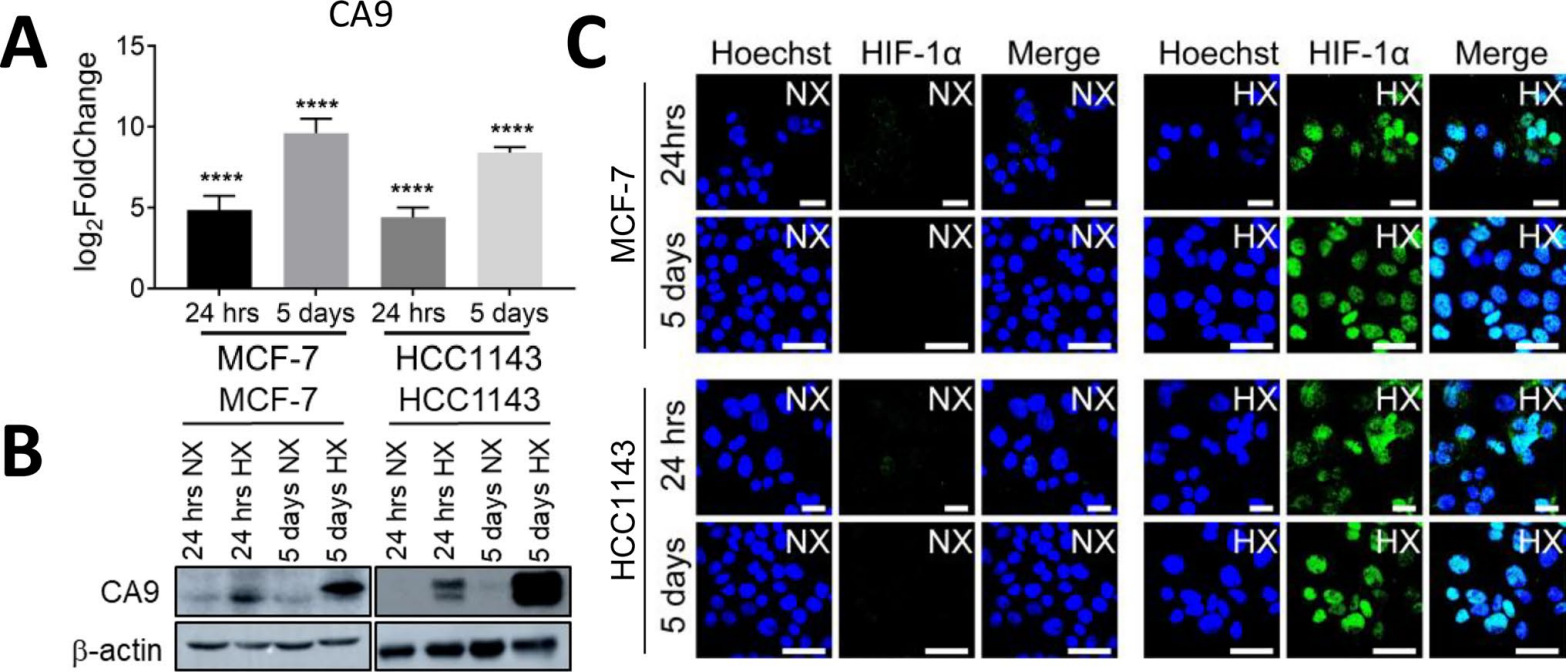
CA9 expression and activation of HIF1α signaling under acute and chronic hypoxia in MCF-7 and HCC1143 cell lines. **A** CA9 RNA expression in four datasets of transcriptome data, including MCF-7 24 h (MCF-7 24 h hypoxia normalized to MCF-7 24 h normoxia), HCC1143 24 h (HCC1143 24 h hypoxia normalized to HCC1143 24 h normoxia), MCF-7 5 days (MCF-7 5 days hypoxia normalized to MCF-7 5 days normoxia), HCC1143 5 days (HCC1143 5 days hypoxia normalized to HCC1143 5 days normoxia). Three biological replicates for each dataset; ****padj < 0.0001. **B, C** CA9 protein expression detected by western blotting **(B)** and Immunofluorescence of HIF1α localization by confocal microscopy **(C)** after 24 h (acute) and 5 days (chronic) incubation under normoxia (NX) and hypoxia (HX) in MCF-7 and HCC1143 cell lines. Blue, Hoechst; Green, HIF1α Ab. One representative experiment of three biological replicates is shown

To characterize the effects of acute and chronic hypoxia in breast cancer cells, we selected two representative human breast cancer cell lines for the luminal (MCF-7) and basal A (HCC1143) subtypes [23]. The cells were exposed to normoxia (21% O_2_) or hypoxia (1% O_2_) for acute (24 h) and chronic (5 days) periods. RNA was collected for transcriptomics analysis using Tempo-Seq high throughput targeted sequencing technology [19]. Using the DESeq2 R package, DEGs in hypoxia when compared to normoxia were identified. To ensure that cells had experienced hypoxic conditions, we analyzed the expression of key hypoxic biomarkers including HIF1α and its target gene, CA9. CA9 mRNA expression was significantly increased in the Tempo-Seq data in response to acute hypoxia and further increased in response to chronic hypoxia for both subtypes (Fig. 1A). This pattern was validated at the protein level by Western blot (Fig. 1B). In agreement, HIF1⍺ immunostaining showed increased accumulation in the nuclei of both cell lines exposed to acute hypoxia and this early, rapid response was not further increased by chronic exposure (Fig. 1C).

### Overview of gene expression patterns in response to acute versus chronic hypoxia

**Fig. 2:**
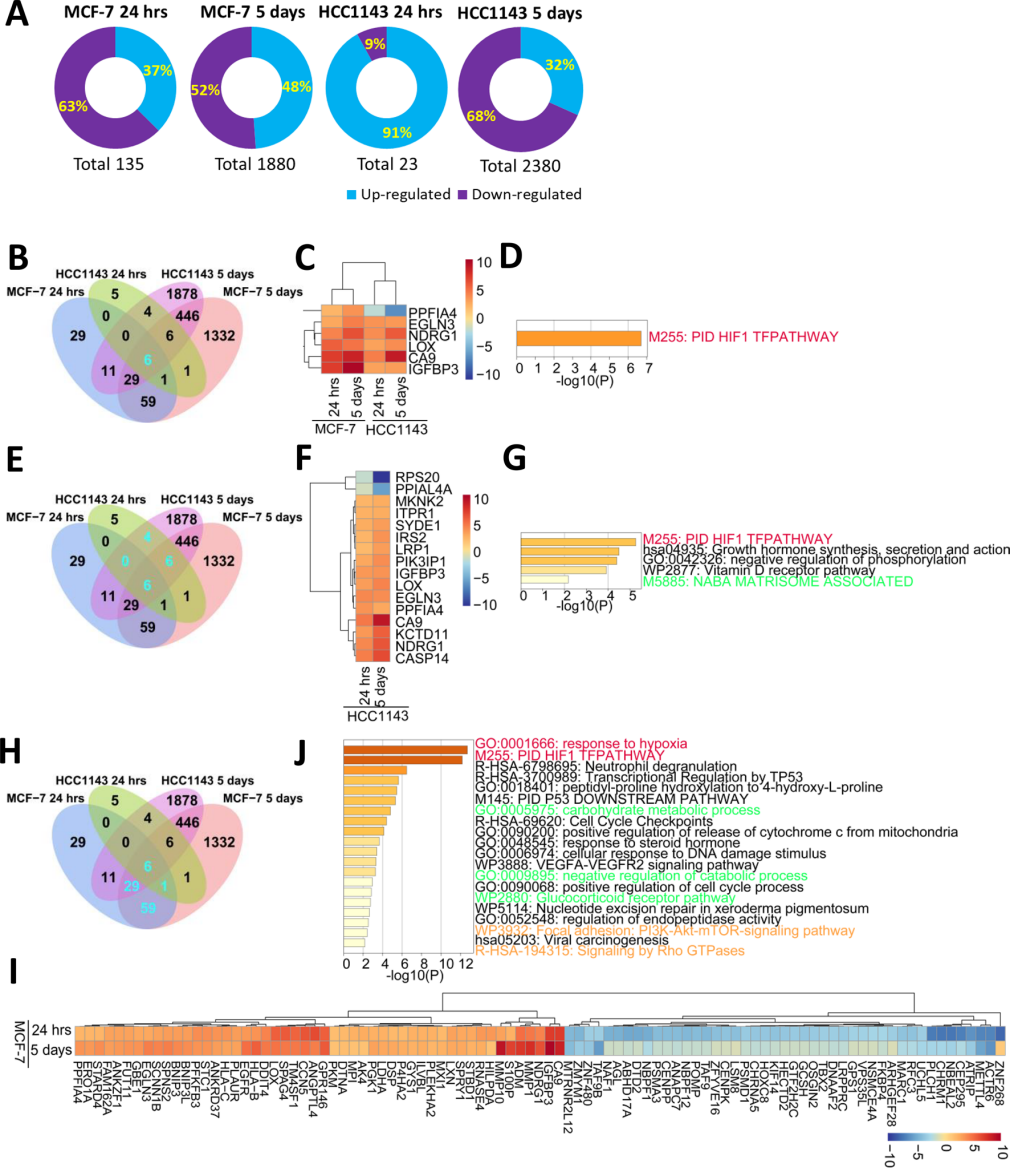
Transcriptome analysis of acute and chronic hypoxia in MCF-7 and HCC1143 cells. **A** Pie charts of up- and down-regulated DEGs in four datasets, including MCF-7 24 h, HCC1143 24 h, MCF-7 5 days and HCC1143 5 days. **B–D** Venn diagram of the four datasets showing six shared DEGs in cyan **(B)** with associated heatmap **(C)**, and top clusters of enriched terms **(D)**. **E–G** Venn diagram of four datasets showing 4 + 6 + 6 shared DEGs for HCC1143 cells 24 h and 5 days hypoxia in cyan **(E)** with associated heatmap **(F)**, and top clusters of enriched terms **(G)**. **H–J** Venn diagram of 4 datasets showing 1 + 6 + 29 + 59 shared DEGs for MCF-7 cells 24 h and 5 days hypoxia in cyan **(H)** with associated heatmap **(I)**, and top clusters **(J)**. Enrichment analysis (**D, G, J**) was done with Metascape. Enriched items associated with HIF signaling, metabolism, and cytoskeleton are labelled in red, green and orange, respectively

Having validated that both cell lines were sensitive to the hypoxic environment, the impact of the duration of exposure was addressed. In both subtypes, chronic hypoxia led to a higher number of DEGs (|log_2_Foldchange|> 1; padj < 0.05) as compared to acute hypoxia (MCF-7, 1880 vs 135 DEGs (Fig. 2A); HCC1143, 4947 vs 23 DEGs). Given the high number of DEGs in the HCC1143 5-day dataset, we used more stringent criteria including |log_2_Foldchange|> 2 and padj < 0.01 and reduced the number of DEGs to 2380 (Fig. 2A). Interestingly, the switch from acute to chronic hypoxia led to a switch from a limited number of predominantly upregulated genes to a large number of predominantly downregulated genes in HCC1143 cells. Only six genes (NDRG1, CA9, IGFBP3, LOX, EGLN3, PPFIA4) overlapped between the four datasets and GO term analysis showed that these were all enriched in the HIF1 pathway (Fig. 2C, D). NDRG1, CA9, IGFBP3, LOX and EGLN3 were up-regulated in all four datasets, whereas PPFIA4 was up-regulated in MCF-7 but down-regulated in HCC1143 cells (Fig. 2C). For HCC1143 cells, 16 genes overlapped between acute and chronic hypoxia, and most of these were upregulated (Fig. 2E, F). These 16 genes were not only enriched in the HIF1α pathway but also in the Growth hormone synthesis, secretion, and action pathway (Fig. 2F, G). For MCF-7 cells, 95 genes overlapped between 24 h and 5 days of hypoxic injury (Fig. 2H), of which 50 genes were upregulated and 44 genes were downregulated (Fig. 2I). These 95 genes were enriched in response to hypoxia, HIF1α pathway, carbohydrate metabolic process and cell cycle checkpoints (Fig. 2J). These data indicated that acute hypoxic stress triggered early adaptation by activation of the HIF1α pathway and chronic hypoxia led to further adaptation, e.g., including proliferation and metabolism.

### Differential response to hypoxia for basal A and luminal cells

**Fig. 3:**
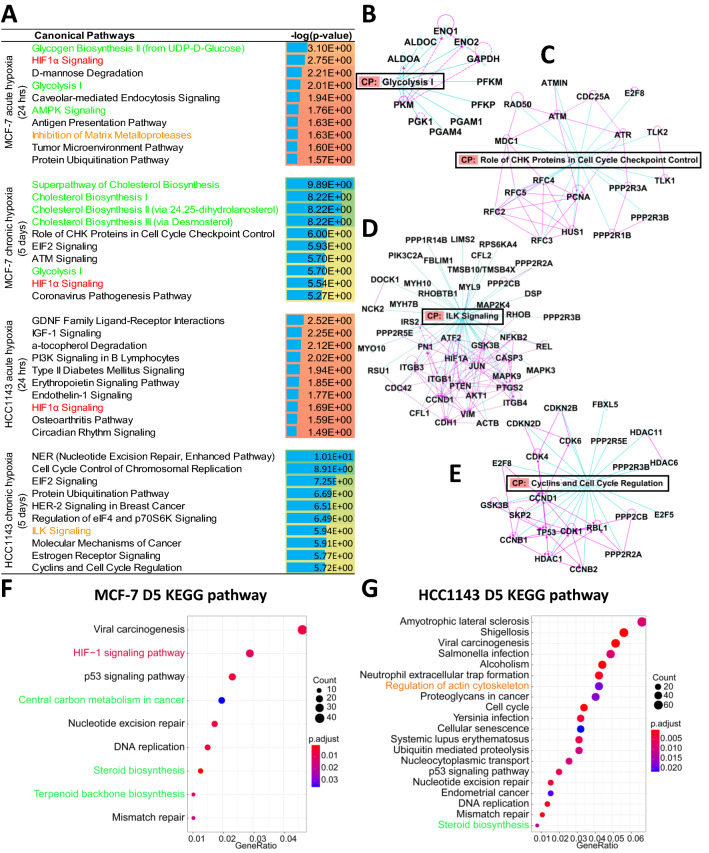
Pathway analysis of acute and chronic hypoxia in MCF-7 and HCC1143 cell lines. **A** Top10 Canonical pathways identified by IPA ranked by *p*-value in acute and chronic hypoxia in MCF-7 and HCC1143 cell lines. The color range from orange to yellow to green reflects –log(*p*-value) from low to high. The length of the blue bar reflects the higher –log(*p*-value). **B–E** Networks with gene interactions indicated by pink lines drawn by IPA for canonical pathways, including Glycolysis **(B)** and Role of CHK protein in cell cycle checkpoint control **(C)** in MCF-7 cells exposed to chronic hypoxia; and ILK signaling **(D)** and Cyclins and cell cycle regulation **(E)** in HCC1143 cells exposed to chronic hypoxia. **F, G** KEGG pathway analysis in chronic hypoxia in MCF-7 **(F)** and HCC1143 **(G)** cells. Pathways associated with HIF signaling (red), metabolism (green), and cytoskeleton (orange) are marked both in IPA (**A**) and KEGG (**F, G**)

We analyzed the DEGs using IPA to map the signaling pathways related to hypoxia. Activation of HIF-1α signaling was a common response in both cell lines exposed to acute hypoxia while cell cycle regulation was affected upon chronic hypoxia also in both cell lines (Fig. 3A, C and E). Yet, several other pathways were differentially affected between the two cell models. Interestingly, in MCF-7 cells exposed to chronic hypoxia changes in metabolic processes including glycolysis and cholesterol biosynthesis were dominant (Fig. 3A and B) whereas these did not appear in HCC1143 cells, where, instead pathways related to the cytoskeleton such as ILK Signaling appeared (Fig. 3A and D). Subsequently, KEGG pathway analysis was used. In agreement with the results obtained using IPA, metabolism was affected in MCF-7 (Fig. 3F) while regulation of the actin cytoskeleton was affected in HCC1143 cells after 5 days of hypoxia (Fig. 3G). These data indicated that MCF-7 and HCC1143 shared an acute activation of the HIF1α pathway and subsequent changes in cell cycle regulation but also showed distinct responses to chronic hypoxia with more metabolic reprogramming in MCF-7 and cytoskeletal adaptation in HCC1143 cells.

### Identification of hypoxia-related hub genes and their clinical relevance

**Fig. 4:**
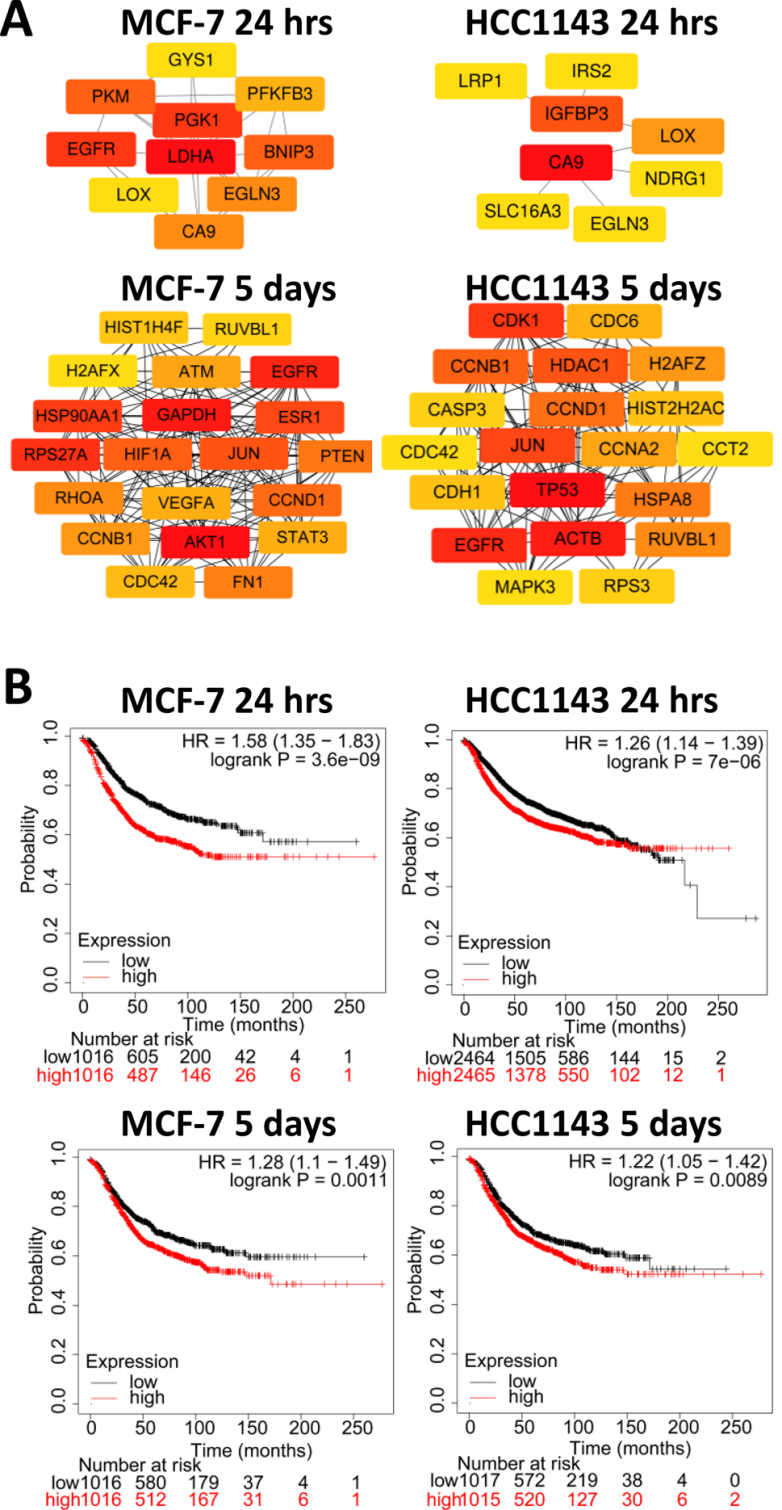
Hub genes of acute and chronic hypoxia in MCF-7 and HCC1143 cell lines. **A** Hub genes were calculated by Degree algorithm using Cytoscape (*cytoHubba*) software, top10 and top20 genes were picked for 24 h (acute) and 5 days (chronic) hypoxia, respectively in MCF-7 and HCC1143 cell lines. The color of the genes from red to yellow is the rank of the genes from top to low. **B** Relapse-free survival (RFS) curves for hub genes drawn on the Kaplan–Meier (KM) plotter platform, for breast cancer patients. Mean expression of hub genes was used. Logrank *p*-value < 0.01 was considered significant for difference between high and low expressors

Cytoscape (*cytoHubba*) was used to identify and select hub genes with the Degree method [24]. Because of the different numbers of DEGs depending on the duration of hypoxia, we selected the top 10 and top 20 genes for acute and chronic hypoxia, respectively, and displayed them in their corresponding network (Fig. 4A). In the acute phase of low oxygen level, hub genes in both cell lines contained several targets of HIF1α signaling, including CA9, IGFBP3, LOX, PFKFB3, PKM, and SLC16A3. In response to chronic hypoxia, hub genes diverged between the two cell lines except for genes related to cell proliferation such as EGFR, cyclin B1 (CCNB1) and cyclin D1 (CCND1). In agreement with earlier pathway analysis (Fig. 3), GAPDH, a key metabolic enzyme in the glycolysis pathway was among the top 10 hub genes in MCF-7; on the other hand ACTB, encoding β actin, was a prominent hub gene in HCC1143. Next, the clinical relevance of the identified hub genes was analyzed using the Kaplan–Meier plotter platform. The mean expression of the four classes of hub genes (associated with acute or chronic hypoxia in MCF-7 or HCC1143) was significantly, negatively associated with relapse-free survival (RFS), with HR > 1.2 and log Rank *p* < 0.01 (Fig. 4B). These findings validated the prognostic value of the newly identified hypoxia-related hub genes in breast cancer patients.

### Chronic hypoxia impairs breast cancer cell proliferation

**Fig. 5:**
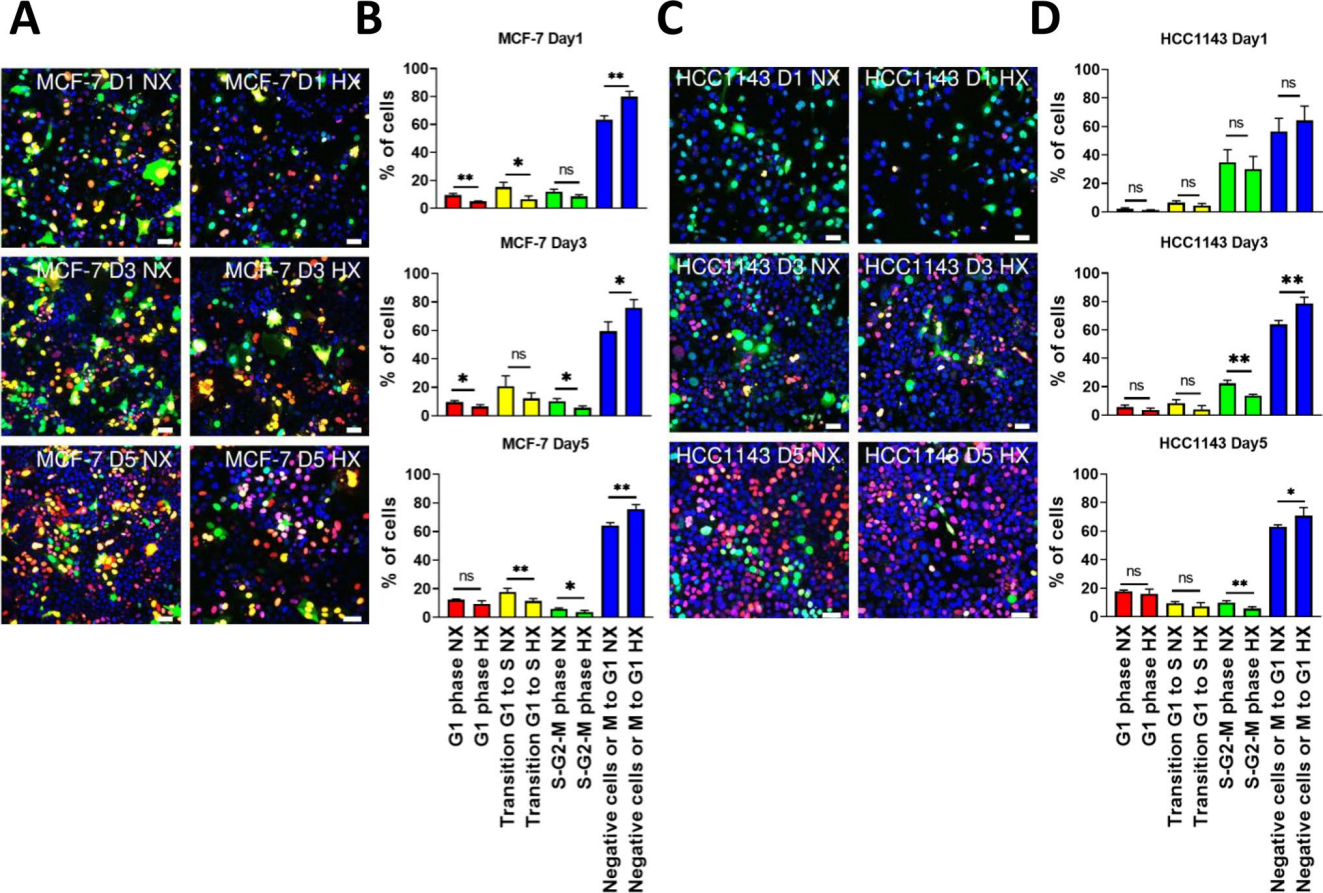
Cell phases in acute and chronic normoxia and hypoxia in MCF-7 and HCC1143 cell lines. **A, C** MCF-7 FUCCI reporter **(A)** and HCC1143 FUCCI reporter cells **(C)** were grown in normoxia (NX) and hypoxia (HX) for 1 day, 3 days and 5 days. Microscopy images show cell cycle phases marked by red (arrested in G1), yellow (transition G1 to S), green (S-G2-M), and blue nuclei (no staining; M-G1). **B, D** Quantification of cell cycle phases in MCF-7 **(B)** and HCC1143 **(D)** by Cellprofiler. Error bars indicate SD for triplicate measurements. **p < 0.01; *p < 0.05; ns, not significant

Our transcriptomics analysis revealed that altered expression of cell cycle-related genes was a common response to acute and chronic hypoxia for both cell lines. We established FUCCI reporter MCF-7 and HCC1143 cell lines to address the impact of hypoxia on the cell cycle. Our previous data showed that cell proliferation was decreased under chronic hypoxia [25]. Using confocal microscopy, distribution across cell cycle phases was quantified at 1-, 3-, and 5-day under normoxia and hypoxia. HCC1143 contained a larger fraction of cells in S-G2-M than MCF-7 and this fraction decreased progressively under normoxia as confluency was reached (Fig. 5A–D). In both cell lines, 3- and 5-day culture in hypoxia led to a stronger reduction in the fraction of cells in S-G2-M. In MCF-7, the fraction of cells in G1-S was also reduced under hypoxia but this was not observed in HCC1143. The apparent reduction in S phase was accompanied by a concomitant increase in unlabeled cells, pointing to an arrest or slower progression in early G1 that was more prominent in hypoxia (Fig. 5).

### Metabolic adaptation of luminal breast cancer cells under chronic hypoxia

**Fig. 6:**
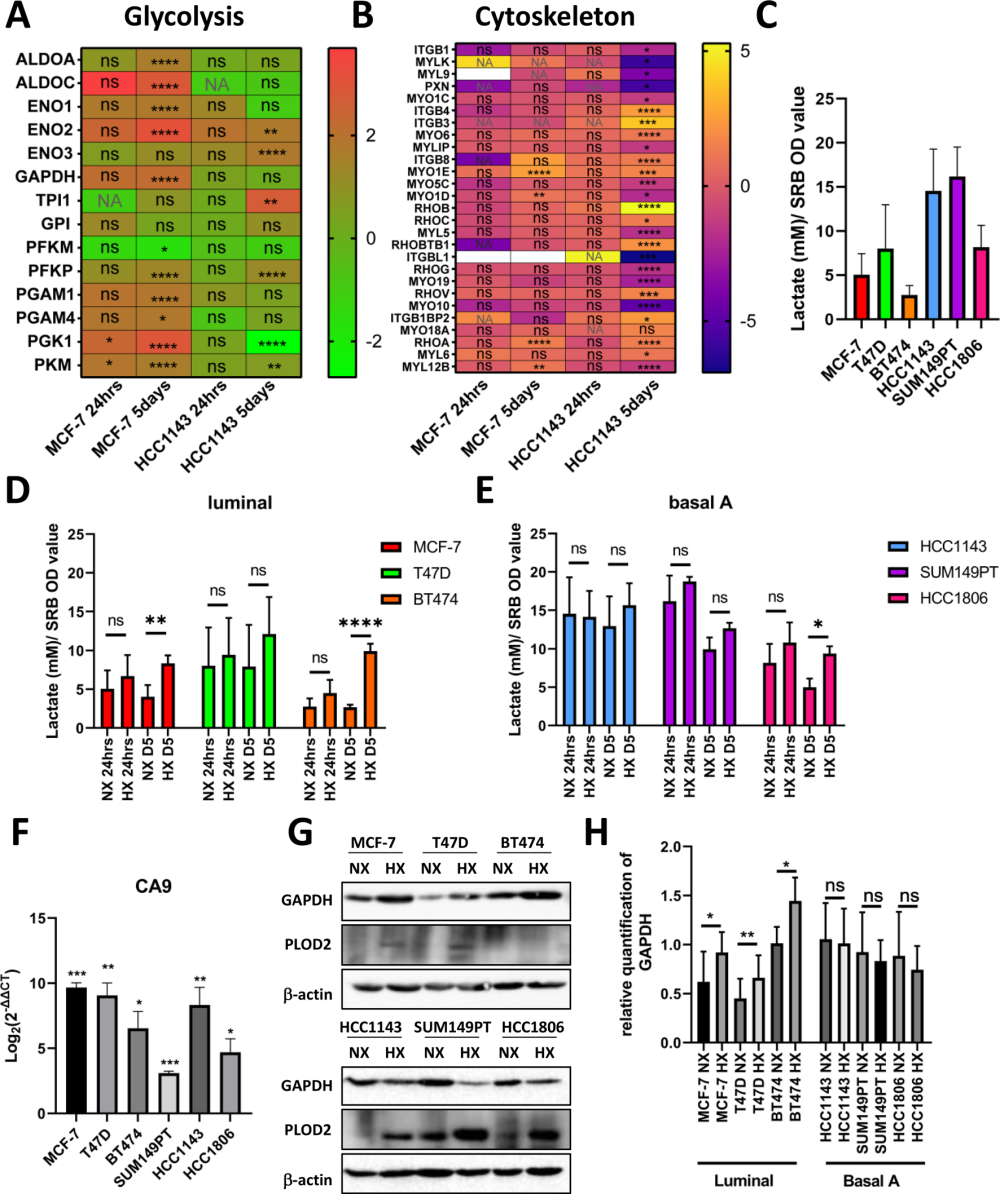
Chronic hypoxia affects metabolism in luminal breast cancer cells. **A, B** Heatmap of genes involved in glycolysis **(A)** and cytoskeleton **(B)** pathways in 24 h (acute) and 5 days (chronic) hypoxia in MCF-7 and HCC1143 cells. NA, not available; ****padj < 0.0001; ***padj < 0.001; **padj < 0.01; *padj < 0.05; ns, not significant. White color means that genes were filtered out in targeted RNA sequencing data after filtering by DESeq2 package in R software. **C** Lactate levels (mM) in 6 cell lines under normoxia normalized to the OD value determined in SRB assay. **D, E** Lactate levels measured in three luminal (MCF-7, T47D, BT474) **(D)** and 3 basal A (HCC1143, SUM149PT, HCC1806) **(E)** breast cancer cell lines under acute normoxia/ hypoxia and chronic normoxia/ hypoxia normalized to the OD value determined in SRB assay. **F** CA9 RNA expression level under hypoxia in luminal and basal A cell lines detected by qRT-PCR. Log_2_(2^(-ΔΔCT)) was calculated by normalizing to normoxia in each cell line. Error bars indicate SD for triplicate measurements. ***p < 0.001; **p < 0.01; *p < 0.05. **G** GAPDH and PLOD2 protein expression detected by Western blot. B-actin serves as loading control. **H** Quantification of GADPH signal normalized to B-actin with Image J. Error bars indicate SD for triplicate measurements. **p < 0.01; *p < 0.05; ns, not significant

Based on the transcriptomics data, chronic hypoxia differentially affected MCF-7 and HCC1143 cells with a predicted effect on glycolysis, particularly in MCF-7 (Figs. 3 and 4). Indeed, several genes related to glycolysis were significantly upregulated in response to chronic hypoxia, uniquely in MCF-7 (Fig. 6A). Conversely, in agreement with the predicted changes of the cytoskeleton organization, several genes associated with the actin cytoskeleton, adhesion, and migration were significantly upregulated in response to chronic hypoxia, uniquely in HCC1143 (Fig. 6B). To address whether this distinction reflected differences between breast cancer subtypes in the response to hypoxia, we first measured lactate levels in three luminal (MCF-7, T47D, and BT474) and three basal A cell lines (HCC1143, SUM149PT, and HCC1806). The average lactate production for all luminal cells under normoxia was lower when compared to the basal A breast cancer cells suggesting that luminal cells were in general less glycolytic (Fig. 6C). Acute hypoxia did not influence lactate production in any of the breast cancer cell lines (Fig. 6D and E). The luminal breast cancer cell lines produced more lactate under chronic hypoxia (although this trend was not significant in T47D), whereas among the basal cell lines, HCC1806 (which had relatively low levels under normoxia) showed a significant increase under hypoxia and the two other basal cell lines (displaying high levels under normoxia) showed no change in lactate levels in response to hypoxia. To further address the apparent trend towards a selective increase in glycolysis in luminal cells, expression of GAPDH, a HIF1⍺ regulated enzyme involved in glycolysis was analyzed. While hypoxia increased CA9 mRNA in all 6 cell lines as expected (Fig. 6F), GAPDH protein levels increased significantly in all three luminal cell lines but were unaffected in the basal A cell line panel (Fig. 6G and H). These data point to increased glycolysis in response to chronic hypoxia particularly in luminal breast cancer cells.

### Increased motility of HCC1143 cells under chronic hypoxia

**Fig. 7:**
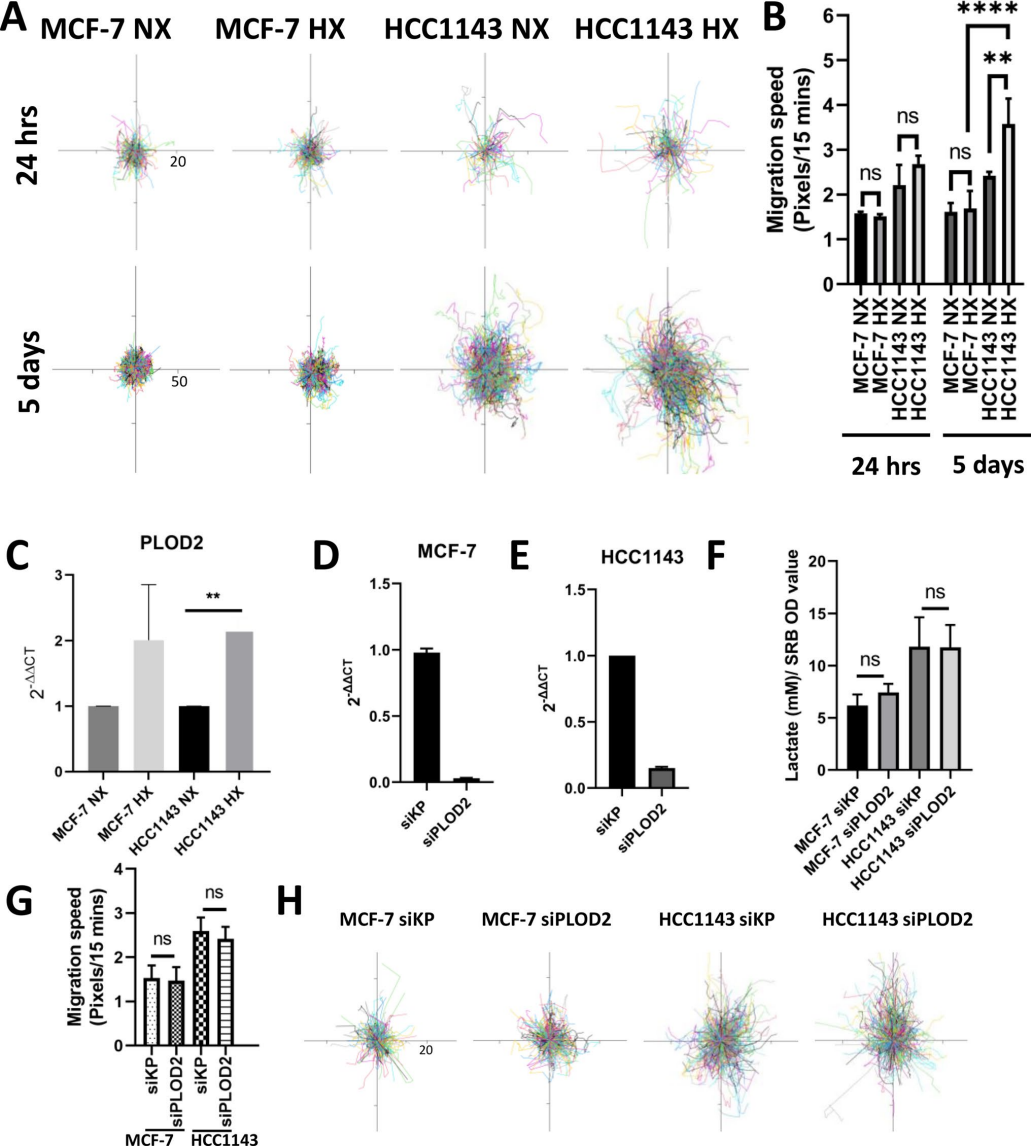
Chronic hypoxia affects cell migration in basal breast cancer cells. **A, B** Cell tracking of MCF-7 and HCC1143 cell lines in acute (24 h) and chronic hypoxia (5 days) **(A)**, and calculated migration speed **(B)**. Error bars indicate SD for triplicate measurements. ****p < 0.0001; **p < 0.01; ns, not significant. The pixels of acute hypoxia are 20 and of chronic hypoxia are 50 for cell tracking. **C** PLOD2 mRNA expression level detected by qRT-PCR in MCF-7 (two biological replicates) and HCC1143 cells (three biological replicates) comparing chronic hypoxia to normoxia. Error bars indicate SD for triplicate measurements. **p < 0.01. **D, E** PLOD2 mRNA expression level in MCF-7 **(D)** and HCC1143 **(E)** cells under chronic hypoxia in presence of PLOD2 siRNA or control “kinasepool” siRNA (siKP). Average and SD from two biological replicates are shown. **F** Lactate levels measured for the indicated cell lines under chronic hypoxia normalized to the OD value determined in SRB assay. **G, H** Migration speed **(G)** and cell tracking (20 pixels) **(H)** analyzed for the indicated cell lines under chronic hypoxia. ns, not significant. Error bars indicate SD for triplicate measurements. ns, not significant

To address the predicted changes in the cytoskeletal organization (Figs. 3, 4 and 6B) in HCC1143 cells, the actin filament organization was analyzed in 3 luminal and 3 basal A cell lines cultured 5 days both under normoxia or hypoxia. In the three basal A cell lines (Fig. S1B), chronic hypoxia led to a more elongated morphology and an increase in stress fiber formation whereas the actin cytoskeleton remained unchanged in the three luminal lines (Fig. S1A). We next asked whether the changes in cytoskeletal morphology affected the migratory behavior of the basal A cells. Indeed, cell migration speed, which was already higher in HCC1143 as compared to MCF-7 cells, was increased in response to chronic hypoxia exclusively in the basal A cells (Fig. 7A and B). PLOD2 is a membrane-bound hydroxylase involved in extracellular matrix crosslinking that has been implicated in cancer cell migration [26] as well as glycolysis [27]. Transcriptomics analysis identified PLOD2 as a DEG induced by chronic hypoxia in MCF-7 whereas it was not significantly changed in HCC1143 cells (not shown). Induction of PLOD2 mRNA in response to chronic hypoxia in MCF-7 was confirmed by qPCR and this showed a similar response in HCC1143 cells (Fig. 7C). Western blot analysis further validated the upregulation of PLOD2 in response to chronic hypoxia in 3 luminal and, more drastically, in 3 basal A cell lines (Fig. 6G). As PLOD2 could connect metabolic adaptation to changes in migration we investigated its role in the response to hypoxia in the luminal and basal A cells. However, siRNA-mediated silencing of PLOD2 in MCF-7 and HCC1143 cells did not affect lactate production nor migration speed under chronic hypoxia (Fig. 7D–H) arguing against such a role. Altogether, these data show that a shared response to hypoxia affecting the HIF1α pathway and cell cycle progression in breast cancer cells, is accompanied by distinct responses to chronic hypoxia with enhanced glycolysis predominantly in luminal cells and a promigratory switch in basal breast cancer cells.

## Discussion

Hypoxia, a common feature in breast cancer can be divided into acute, chronic, and intermittent hypoxia [10, 28]. Thus far, research has largely focused on acute hypoxia when studying the effect of oxygen deprivation in breast cancer cells [29–32]. Yet, cells may adapt differently to the dynamic hypoxic environment, by changing either their metabolism [33], decreasing cell proliferation [34], or becoming more invasive [6].It was recently shown that hypoxia negatively associates with immune activity in TNBC and HER2^+^, but not in luminal breast cancer [35]. However, there is still a limited number of studies investigating the differential response of breast cancer cells to acute and chronic hypoxia [36, 37].

In this study, we performed high throughput targeted RNA sequencing (TempO-Seq) of luminal (MCF-7) and basal A (HCC1143) breast cancer cells cultured either 24 h or 5-day under hypoxic conditions. In line with the transcriptomics data, both FUCCI MCF7 and HCC1143 cell lines showed a reduced fraction in the S-G2-M phase in chronic hypoxia and HCC1143 cells were arrested in early G1 phase. The cell cycle is a vital process that is influenced by cell shape [38], cytoskeletal tension [39], and metabolism [40], which was clearly different between the two cell lines and yet the impact of hypoxia on cell cycle phase distribution was similar for both of them.

Cancer cells produce energy by glycolysis instead of oxidative phosphorylation and our findings are in agreement with earlier studies showing that glycolysis is more prominent in basal A cells than in luminal cells [41, 42]. We find that chronic hypoxia induces glycolysis through upregulation of GAPDH in luminal cells but has little effect on basal A cells in this respect [43, 44].

In basal A cells only, chronic hypoxia stimulates pathways associated with actin cytoskeletal organization and migration such as Rho GTPase signaling which is in line with previous findings [43–49]. Even though, the gene ACTB was upregulated at mRNA level after chronic hypoxia, we did not measure any change at protein level. On the other hand, chronic hypoxia induced a more mesenchymal phenotype as seen by the increased number of stress fibers suggesting differential Rho activity and focal adhesion turnover. Altogether, these data suggest that the basal A subtype may acquire more aggressive features during prolonged hypoxic insult leading to a worst prognosis for the cancer patients. Accordingly, PLOD2, a well-known risk gene for breast cancer patients [27, 45], as it stimulates EMT in cancer [26, 46] is triggered by hypoxia in both luminal cells and more abundantly in basal A cells in our study.

PLOD2 also contributes to glycolysis under hypoxia [47–50]. Despite its significant upregulation, we do not identify a functional role of PLOD2 in hypoxia-stimulated glycolysis or cell migration. Given its role in collagen cross-linking, its pro-migratory effect may be observed especially in 3D culture conditions.

In conclusion, we studied different subtypes of breast cancer cell lines under acute and chronic hypoxia. Chronic hypoxia mainly affects the cytoskeleton in basal A cells and metabolism in luminal cells, which is further confirmed by higher cell migration speed and more F-actin stress fibers in basal A cells and by more lactate productivity and increased GADPH in luminal cells. Our findings indicate distinct responses of luminal-like and basal-like breast cancer cells to hypoxia, which could provide further insights for distinct developing distinct therapeutic strategies for treating different hypoxic breast cancer subtypes.

## Supplementary Information

Below is the link to the electronic supplementary material.

Supplementary file1 (PDF 323 KB)**Fig. S1** Chronic hypoxia affects actin cytoskeleton mainly in basal A breast cancer cells. **A, B **Phalloidin (red) and Hoechst (blue) staining of 3 luminal (MCF-7, T47D, BT474) **(A)** and 3 basal A (SUM149PT, HCC1806, HCC1143) **(B)** breast cancer cell lines grown under normoxia (NX) and hypoxia (HX) for 5 days.

## Data Availability

TempO-seq data supporting the results of this article will be available in the BioStudies database from EMBL-EBI. chronic data set: https://www.ebi.ac.uk/biostudies/arrayexpress/studies/E-MTAB-12233?key=5b1f9227-fe06-44b4-8909-55bb3ecf7a33. acute data set: https://www.ebi.ac.uk/biostudies/arrayexpress/studies/E-MTAB-12234?key=06b3e420-93c3-4fec-86dc-11c2d688e71c.

## References

[1] Azamjah N, Soltan-Zadeh Y, Zayeri F (2019). Global trend of breast cancer mortality rate: a 25-year study. Asian Pac J Cancer Prev: APJCP.

[2] Mosier JA, Schwager SC, Boyajian DA (2021). Cancer cell metabolic plasticity in migration and metastasis. Clin Exp Metas.

[3] Hanahan D (2022). Hallmarks of cancer: new dimensions. Cancer Discov.

[4] Al Tameemi W, Dale TP, Al-Jumaily RMK (2019). Hypoxia-modified cancer cell metabolism. Front Cell Dev Biol.

[5] Dekker Y, Le Dévédec SE, Danen EHJ (2022). Crosstalk between hypoxia and extracellular matrix in the tumor microenvironment in breast cancer. Genes.

[6] Nagelkerke A, Bussink J, Mujcic H (2013). Hypoxia stimulates migration of breast cancer cells via the PERK/ATF4/LAMP3-arm of the unfolded protein response. Breast Cancer Res.

[7] Tam SY, Wu VWC, Law HKW (2020). Hypoxia-induced epithelial-mesenchymal transition in cancers: HIF-1α and be. Front Oncol.

[8] Lin Q, Cong X, Yun Z (2011). Differential hypoxic regulation of hypoxia-inducible factors 1alpha and 2alpha. Mol Cancer Res.

[9] Muz B, La Puente P, de, Azab F, (2015). The role of hypoxia in cancer progression, angiogenesis, metastasis, and resistance to therapy. Hypoxia (Auckland, NZ).

[10] Saxena K, Jolly MK (2019). Acute vs chronic vs cyclic hypoxia: their differential dynamics, molecular mechanisms, and effects on tumor progression. Biomolecules.

[11] Bayer C, Vaupel P (2012). Acute versus chronic hypoxia in tumors: controversial data concerning time frames and biological consequences. Strahlentherapie und Onkologie.

[12] Liu Q, Palmgren VAC, Danen EH (2022). Acute vs chronic vs intermittent hypoxia in breast cancer: a review on its application in in vitro research. Mol Biol Rep.

[13] Ji W, Wang L, He S (2018). Effects of acute hypoxia exposure with different durations on activation of Nrf2-ARE pathway in mouse skeletal muscle. PloS One.

[14] Reiterer M, Colaço R, Emrouznejad P (2019). Acute and chronic hypoxia differentially predispose lungs for metastases. Sci Rep.

[15] Taube JH, Herschkowitz JI, Komurov K (2010). Core epithelial-to-mesenchymal transition interactome gene-expression signature is associated with claudin-low and metaplastic breast cancer subtypes. Proc Natl Acad Sci USA.

[16] Wu Y, Sarkissyan M, Vadgama JV (2016). Epithelial-mesenchymal transition and breast cancer. J Clin Med.

[17] Takatani-Nakase T, Matsui C, Hosotani M (2022). Hypoxia enhances motility and EMT through the Na+/H+ exchanger NHE-1 in MDA-MB-231 breast cancer cells. Exp Cell Res.

[18] Kierans SJ, Taylor CT (2021). Regulation of glycolysis by the hypoxia-inducible factor (HIF): implications for cellular physiology. J Physiol.

[19] Yeakley JM, Shepard PJ, Goyena DE (2017). A trichostatin A expression signature identified by TempO-Seq targeted whole transcriptome profiling. PloS One.

[20] Carpenter AE, Jones TR, Lamprecht MR (2006). Cell profiler: image analysis software for identifying and quantifying cell phenotypes. Genome Biol.

[21] Wink S, Hiemstra S, Huppelschoten S (2014). Quantitative high content imaging of cellular adaptive stress response pathways in toxicity for chemical safety assessment. Chem Res Toxicol.

[22] Zhou Y, Zhou B, Pache L (2019). Metascape provides a biologist-oriented resource for the analysis of systems-level datasets. Nat Commun.

[23] Dai X, Cheng H, Bai Z (2017). Breast cancer cell line classification and its relevance with breast tumor subtyping. J Cancer.

[24] Xi Y, Liu J, Shen G (2022). Low expression of IGFBP4 and TAGLN accelerate the poor overall survival of osteosarcoma. Sci Rep.

[25] Liu Q, van der Stel W, van der Noord VE (2022). Hypoxia triggers TAZ phosphorylation in basal A triple negative breast cancer cells. Int J Mol Sci.

[26] Gilkes DM, Bajpai S, Wong CC (2013). Procollagen lysyl hydroxylase 2 is essential for hypoxia-induced breast cancer metastasis. Mol Cancer Res.

[27] Du W, Liu N, Zhang Y (2020). PLOD2 promotes aerobic glycolysis and cell progression in colorectal cancer by upregulating HK2. Biochem Cell Biol.

[28] Peir CHF, Encina JA, Perez MM (2019). The role of hypoxia-induced factor 1 in breast cancer. JCMT.

[29] Azimi I, Petersen RM, Thompson EW (2017). Hypoxia-induced reactive oxygen species mediate N-cadherin and SERPINE1 expression, EGFR signalling and motility in MDA-MB-468 breast cancer cells. Sci Rep.

[30] Cooper C, Liu G-Y, Niu Y-L (2004). Intermittent hypoxia induces proteasome-dependent down-regulation of estrogen receptor alpha in human breast carcinoma. Clin Cancer Res.

[31] Han J, Li J, Ho JC (2017). Hypoxia is a key driver of alternative splicing in human breast cancer cells. Sci Rep.

[32] Karlenius TC, Shah F, Di Trapani G (2012). Cycling hypoxia up-regulates thioredoxin levels in human MDA-MB-231 breast cancer cells. Biochem Biophys Res Commun.

[33] Takeda K, Arase S, Takahashi S (1988). Side effects of topical corticosteroids and their prevention. Drugs.

[34] Tang K, Zhu L, Chen J (2021). Hypoxia promotes breast cancer cell growth by activating a glycogen metabolic program. Can Res.

[35] Ma S, Zhao Y, Lee WC (2022). Hypoxia induces HIF1α-dependent epigenetic vulnerability in triple negative breast cancer to confer immune effector dysfunction and resistance to anti-PD-1 immunotherapy. Nat Commun.

[36] Jarman EJ, Ward C, Turnbull AK (2019). HER2 regulates HIF-2α and drives an increased hypoxic response in breast cancer. Breast Cancer Res.

[37] Stiehl DP, Bordoli MR, Abreu-Rodríguez I (2012). Non-canonical HIF-2α function drives autonomous breast cancer cell growth via an AREG-EGFR/ErbB4 autocrine loop. Oncogene.

[38] Clark AG, Paluch E (2011). Mechanics and regulation of cell shape during the cell cycle. Results Probl Cell Differ.

[39] Huang S, Chen CS, Ingber DE (1998). Control of cyclin D1, p27(Kip1), and cell cycle progression in human capillary endothelial cells by cell shape and cytoskeletal tension. Mol Biol Cell.

[40] Icard P, Fournel L, Wu Z (2019). Interconnection between metabolism and cell cycle in cancer. Trends Biochem Sci.

[41] Masson N, Ratcliffe PJ (2014). Hypoxia signaling pathways in cancer metabolism: the importance of co-selecting interconnected physiological pathways. Cancer Metabol.

[42] Farhadi P, Yarani R, Valipour E (2021). Cell line-directed breast cancer research based on glucose metabolism status. Biomed Pharmacother.

[43] Zhu X, Jin C, Pan Q (2021). Determining the quantitative relationship between glycolysis and GAPDH in cancer cells exhibiting the Warburg effect. J Biol Chem.

[44] Hjerpe E, Egyhazi Brage S, Carlson J (2013). Metabolic markers GAPDH, PKM2, ATP5B and BEC-index in advanced serous ovarian cancer. BMC Clin Pathol.

[45] Xu F, Guan Y, Xue L (2020). The effect of a novel glycolysis-related gene signature on progression, prognosis and immune microenvironment of renal cell carcinoma. BMC Cancer.

[46] Du H, Pang M, Hou X (2017). PLOD2 in cancer research. Biomed Pharmacother.

[47] Xu F, Zhang J, Hu G (2017). Hypoxia and TGF-β1 induced PLOD2 expression improve the migration and invasion of cervical cancer cells by promoting epithelial-to-mesenchymal transition (EMT) and focal adhesion formation. Cancer Cell Int.

[48] Xu Y, Zhang L, Wei Y (2017). Procollagen-lysine 2-oxoglutarate 5-dioxygenase 2 promotes hypoxia-induced glioma migration and invasion. Oncotarget.

[49] Wan J, Qin J, Cao Q (2020). Hypoxia-induced PLOD2 regulates invasion and epithelial-mesenchymal transition in endometrial carcinoma cells. Genes Genomics.

[50] Gilkes DM, Bajpai S, Chaturvedi P (2013). Hypoxia-inducible factor 1 (HIF-1) promotes extracellular matrix remodeling under hypoxic conditions by inducing P4HA1, P4HA2, and PLOD2 expression in fibroblasts. J Biol Chem.

