# Supplementary figures and images for: Differential response of luminal and basal breast cancer cells to acute and chronic hypoxia
Source: Breast Cancer Res Treat. 2023 Feb 24;198(3):583–96. doi: 10.1007/s10549-023-06863-w (PMC10036440; doi:10.1007/s10549-023-06863-w)

**A**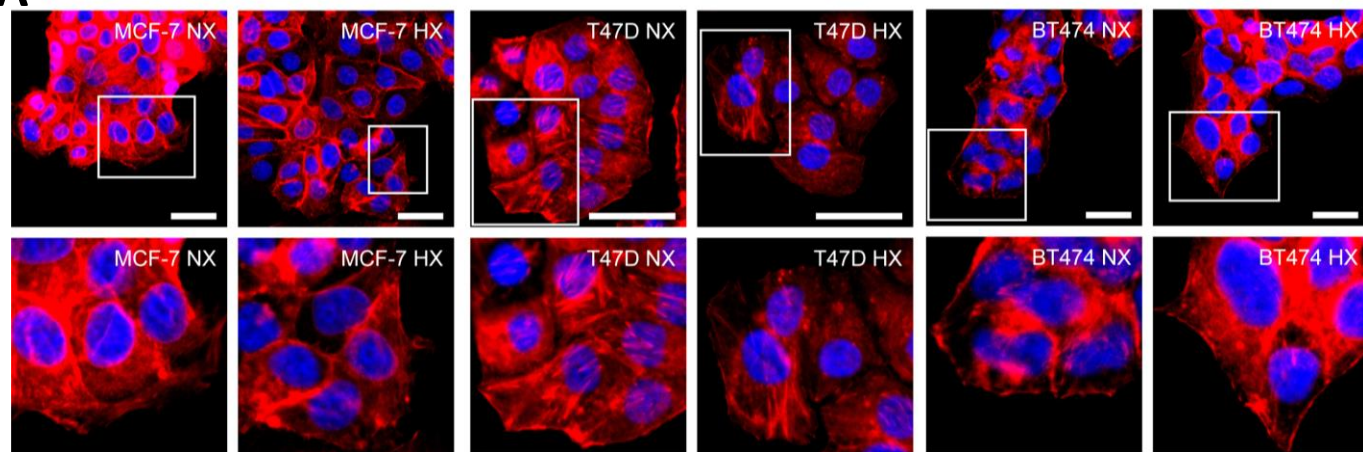**B**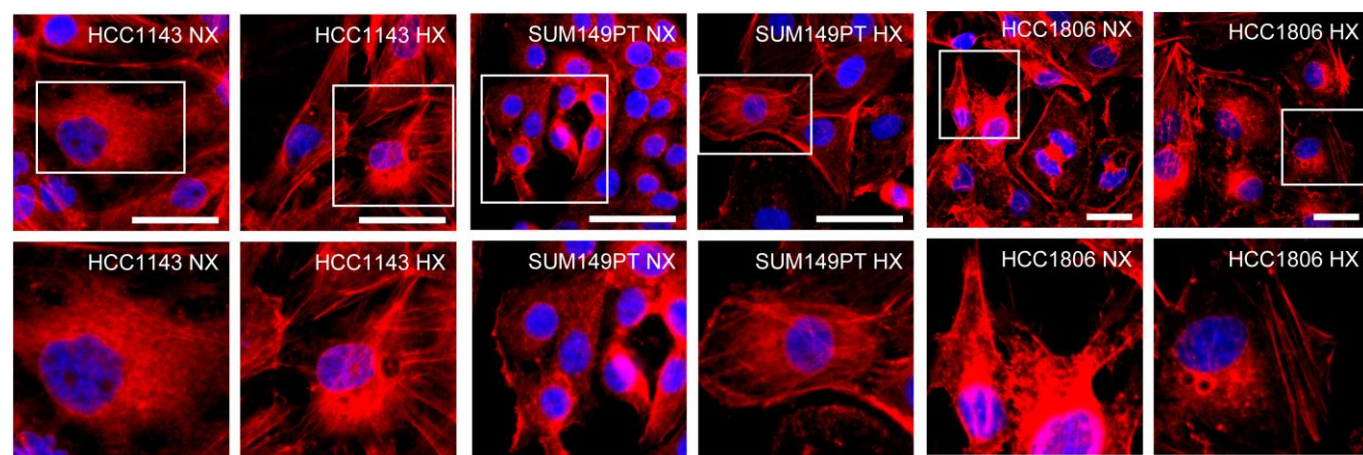

Figure S1

Supplement: Supplementary file 1 — Supplementary file1 (PDF 323 KB)Fig. S1 Chronic hypoxia affects actin cytoskeleton mainly in basal A breast cancer cells. A, B Phalloidin (red) and Hoechst (blue) staining of 3 luminal (MCF-7, T47D, BT474) (A) and 3 basal A (SUM149PT, HCC1806, HCC1143) (B) breast cancer cell lines grown under normoxia (NX) and hypoxia (HX) for 5 days. [file 10549_2023_6863_MOESM1_ESM.pdf]
